# Needs led research: ensuring relevant research in two PhD projects within maternity care

**DOI:** 10.1186/s40900-024-00627-6

**Published:** 2024-09-12

**Authors:** Kristin Jerve Aanstad, Kjersti Engen Marsdal, Ellen Blix, Anne Kaasen, Mirjam Lukasse, Ingvil Krarup Sørbye, Ida Svege

**Affiliations:** 1 Faculty of Health Sciences, Department of Nursing and Health Promotion, Oslo Metropolitan University, Pilestredet 32, Oslo, 0167 Norway; 2 Division of Obstetrics and Gynecology, Oslo University Hospital, Oslo, Norway; 3 Department of Nursing and Social Sciences, Institute of Nursing and Health Sciences, University of South-Eastern Norway, Campus Vestfold, Tønsberg, Norway; 4 Institute of Clinical Medicine, Faculty of Medicine, University of Oslo, Oslo, Norway; 5 Nordic Institute for Studies in Innovation, Education and Research (NIFU), Oslo, Norway

**Keywords:** Needs led research, Knowledge user involvement, Priority setting, Maternity care

## Abstract

**Background:**

There has been a growing concern regarding research waste and the mismatch between conducted research and the research needs of knowledge users. The Needs Led Research (NLR) approach is proposed as an effective method to ensure that research address actual evidence gaps that are relevant to the users of the knowledge. By search and reviewing literature and involving knowledge users, NLR aims to identify, verify, and prioritize research needs. This paper describes and compares the implementation of the NLR approach in two separate PhD projects within maternity care, and addresses the challenges encountered throughout the processes, aiming to offer valuable insights for future NLR initiatives.

**Methods:**

The NLR processes consisted of four phases: (1) defining the scope (2) identifying and verifying research needs (3) prioritizing research needs and (4) designing the PhD projects. Literature searches were conducted during Phase 2, while knowledge user involvement took place in Phases 2 and 3. The knowledge user involvement, at a co-thinker and advocatory level, included knowledge user groups and surveys. Project groups, who were responsible for all decision-making, conducted Phases 1 and 4. The scopes of the PhD projects were labor induction (NLR-LINO) and fetal monitoring in low-risk deliveries (NLR-LISTEN).

**Results:**

In NLR-LINO, 17 research needs were identified and verified as actual evidence gaps relevant for the knowledge users. Among these, ten were rated as “very important” by a majority of the 322 survey respondents. The aim of the PhD LINO project was defined as “To investigate whether outpatient induction of labor is beneficial in a Norwegian setting.” In NLR-LISTEN, seven research needs were identified and verified as actual evidence gaps relevant for the knowledge users. These were prioritized by 466 survey respondents, and the aim of the PhD LISTEN project was defined as “To investigate the methods used for fetal monitoring in low-risk deliveries in Norway and evaluate adherence to evidence-based practice while also exploring potential reasons for any deviations.”

**Conclusions:**

This paper shows that the NLR is a viable approach for prioritizing research. The findings highlight the impact of the initial scope on subsequent phases and emphasize the importance of pragmatic decision-making throughout the process. However, it is crucial to acknowledge that NLR requires dedicated resources, and if integrated into PhD projects, additional time and training should be allocated accordingly.

**Supplementary Information:**

The online version contains supplementary material available at 10.1186/s40900-024-00627-6.

## Abstract (plain language summary)

This paper addresses the issue of research waste and the mismatch between conducted research and the actual needs of the users of the knowledge. The Needs Led Research (NLR) approach is proposed as an effective method for identifying and prioritizing research needs that are relevant to knowledge users and have not been adequately addressed by existing research. The paper specifically examines the implementation of the NLR approach in two separate PhD projects within the field of maternity care, aiming to provide valuable insights for future NLR initiatives. The NLR process consists of four phases: defining the scope, identifying and verifying research needs, prioritizing those needs, and designing the PhD projects. Literature searches were conducted during Phase 2 to identify existing evidence gaps in the research literature. Knowledge users were involved in both the identification and prioritization of research needs in Phases 2 and 3 through surveys and group discussions. In conclusion, this paper shows that NLR is a viable approach for prioritizing research and ensuring its relevance to the users of the knowledge. It emphasizes the importance of defining a clear scope and making pragmatic decisions throughout the process. However, it also highlights the need for dedicated resources and additional time and training when integrating the NLR approach into PhD projects.

**Supplementary Information:**

The online version contains supplementary material available at 10.1186/s40900-024-00627-6.

## Background

In 2009, Chalmers and Glasziou suggested that 85% of health research was wasted, partly because researchers chose the wrong questions to address in their research [1]. The Lancet continued the discussion in 2014 with a series of papers on increasing the value of health research and reducing avoidable waste [2]. One of the papers focused on potential research waste caused by overlooking existing knowledge and ignoring the needs of the users of the research [3]. These issues have also been noted by others [4–7]. When a review of existing evidence is not performed, there is a risk of repeating studies where the research questions are already answered, resulting in both wasted resources and exposing patients for unnecessary potential side effects [3, 8, 9]. The inclusion of knowledge users, defined as individuals who might use or be affected by the knowledge generated, in prioritizing research ensures that new research addresses questions that are relevant [3, 8, 10, 11]. The mismatch between patients’ and clinicians’ research needs and the research conducted has been increasingly recognized by policymakers and research funders, leading to a growing emphasis on involving knowledge users in research prioritizing and planning [11–14].

To address the challenge of research waste, various approaches to prioritize health research have been suggested [15–18]. While a wide range of methods and approaches are available, there is no consensus on the preferred methods [15, 19]. The James Lind Alliance (JLA) is an initiative that combines literature reviews and knowledge user involvement in priority-setting partnerships, making lists of the top ten priorities for guidance of future research in various areas [20]. While the JLA process is time consuming and often conducted at an overarching level, smaller-scale projects may require adapting priority processes to serve its purpose and align with the available resources [21]. One such approach, partly inspired by the JLA, is Needs Led Research (NLR). The NLR is a term agreed upon by the authors of the paper “The Bridge Building Model - connecting evidence-based practice, research public involvement and needs led research” [22]. The NLR process aims to ensure that research projects address actual evidence gaps that are relevant to knowledge users by identifying, verifying, and prioritizing research needs through literature reviews and knowledge user involvement.

Maternity care is an that is area well suited for research prioritization processes. While childbirth can be considered primarily a physiological and potentially medical event, it additionally holds immense significance for the women and their families, encompassing a complex process of personal, social and cultural aspects [23]. This is reflected in the World Health Organization’s recommendations for care during childbirth, emphasizing the importance of ensuring not only ensuring the survival of women and their babies but also a positive childbirth experience [24]. The complexity of pregnancy and childbirth can give rise to a wide range of perspectives, resulting in variations in opinions among different groups of stakeholders regarding the prioritization of research [6]. While an increasing number of prioritization processes are being implemented in maternity care research, labor induction and fetal monitoring have yet to be addressed [18, 25]. In addition, there are few published examples of how research prioritization can be implemented in a PhD setting. The lack of published experiences can pose a barrier to adopting such practices [26, 27]. Thus, the objective of this paper is to describe and compare the implementation of the NLR approach, as described by Ormstad et al. [22], in two separate PhD projects within the field of maternity care. Additionally, we address the challenges encountered throughout the NLR processes, with the aim of offering valuable insights for future NLR initiatives.

## Methods

### Setting

The two separate NLR processes described in this paper are part of two different PhD projects within the field of maternity care: The Labor Induction in Norway study (LINO) and the Low-risk Intrapartum Fetal Monitoring study (LISTEN). The two NLR processes are referred to as NLR-LINO and NLR-LISTEN, respectively. Both NLR-LINO and NLR-LISTEN were part of the Bridgebuilder Initiative at the Faculty of Health Sciences, Oslo Metropolitan University, Norway [28]. This initiative aimed to develop and apply NLR processes for PhD projects based on the principles outlined in the paper “The Bridge Building Model - connecting evidence-based practice, research public involvement and needs led research” by Ormstad et al. [22]. Ten PhD students, including KEM and KJA, entered the PhD program based on preliminary project plans and objectives, without a detailed project protocol or specified research questions. Throughout their initial part of their PhD engagement, from August 2019 to May 2020, they employed various approaches to design their PhD projects based on NLR principles [22, 29–31]. The PhD students in the Bridgebuilder Initiative held five-year scholarships, with 60% of the time dedicated to research and 40% allocated to mandatory duties related to clinical practice.

### The NLR process

The aim of each NLR process was to ensure that the PhD project addressed actual evidence gaps relevant to knowledge users, through literature searches and knowledge user involvement [22]. While inspired by the JLA, the NLR processes were notably smaller and constrained by time and resources [20, 22]. As a result, we made substantial and pragmatic adjustments to the JLA methodology. Unlike the JLA’s aim of creating a top ten list of research questions within a scope, our aim was to define and design our PhD projects [20, 22]. Each NLR process started with an initial plan that was continually adjusted, integrating input from previous phases of the process, as well as advice from supervisors and colleagues in the Bridgebuilder Initiative.

**Fig. 1:**
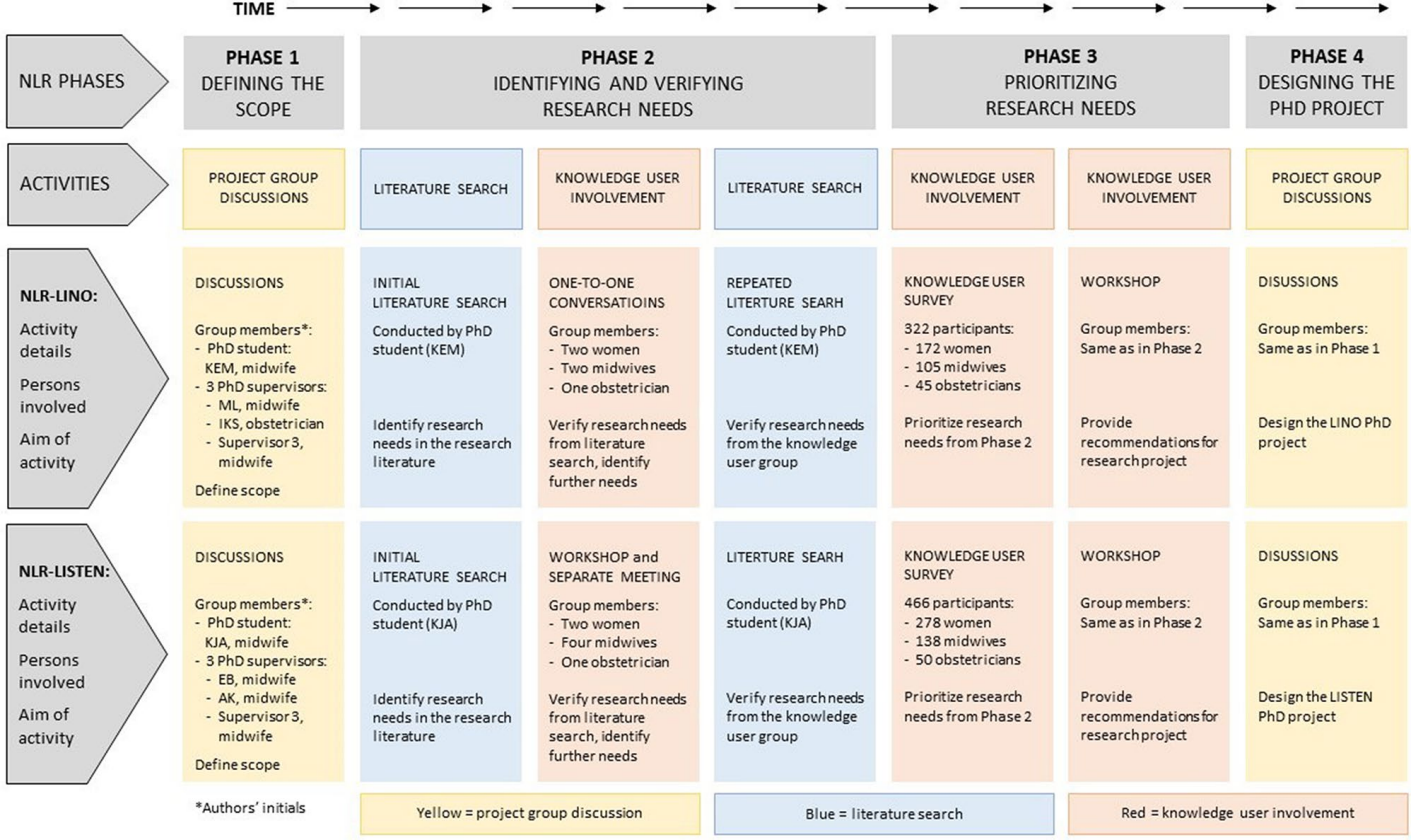
Phases, activities, groups, and knowledge users involved in the NLR processes

The NLR processes of LINO and LISTEN are outlined in Fig. 1 and described in further detail below. Two groups were established for each NLR process: a project group, comprising the respective PhD student and her supervisors, and a knowledge user group. The project groups made the final decisions for the studies, while the knowledge user groups were at a co-thinker and advocatory level [32]. The NLR-processes included four phases, as described by Ormstad et al. [22]: (1) defining the scope (2) identifying and verifying research needs (3) prioritizing research needs and (4) designing the PhD projects. Phases 1 and 4 took place within the project groups, while the knowledge user involvement was conducted in Phases 2 and 3. The literature searches were performed in Phase 2. The phases could overlap, especially Phases 2 and 3.

### Knowledge users

Knowledge users are defined as persons who could use the research results to make informed decisions or who could or be affected by the knowledge generated [10, 11]. This includes but is not limited to, clinicians, patients and their caregivers, patient organizations, decision- and policy makers and researchers. In both NLR-LINO and NLR-LISTEN, the primary knowledge users were defined as women, midwives, and obstetricians, as they were considered to utilize or be most affected by the studies’ findings. In this paper, the term “women” refers to those who have used or may use maternity care services.

### **Knowledge user groups**

A separate knowledge user group was established for each NLR process, for engagement in Phases 2 and 3. Members were recruited using a convenience sampling strategy. In NLR-LINO, one woman was recruited by reaching out to a maternal mental health organization via email [33]. The other woman was approached via email because of her strong interest in maternal health via social media. They had both given birth within the last five years. The two midwives and the obstetrician in the NLR-LINO knowledge user group worked at the same maternity care unit at Oslo University Hospital, with 10–20 years of experience. In NLR-LISTEN, posters were displayed at three maternal and child health centers in Oslo to recruit women, but this did not yield any contacts. Hence, midwives at Oslo University Hospital acted as intermediaries by asking women for permission to share their contact information with the PhD student. This led to the recruitment of two women, who had both given birth within the last six months. The four midwives and the obstetrician in the group all worked at the same hospital, but at three different maternity care units. Their work experience ranged from one to 20 years. All knowledge user group members in both NLR-LINO and NLR-LISTEN were cisgender individuals. No payment was provided for participation, but travel expenses were refunded. Midwives and obstetricians in NLR-LINO participated in the knowledge user group activities during their working hours.

### Phase 1 defining the scope

The topic for each project, with preliminary scopes, was determined prior to the NLR-processes, based on the research interest of the members of the project groups. To delimit and define the.

scope, an informal review of the key literature within the topic was conducted, along with input from spontaneous discussions with midwives and obstetricians in maternity care units and within the project groups. The scope of NLR-LINO was defined as “labor induction,” and the scope of NLR-LISTEN was defined as “fetal monitoring in low-risk deliveries” (see Table 1).

**Table 1:** Brief introduction to the scopes of the two PhD projects

Labor Induction in Norway (LINO)	Low-Risk Intrapartum Fetal Monitoring (LISTEN)
• In most pregnancies, labor starts spontaneously. However, inducing labor is sometimes preferred due to maternal or fetal well-being. In Norway, nearly 30% of labors are induced [34]. • The most common indications for inducing labor in Norway include post-term pregnancy, premature rupture of membranes, preeclampsia/hypertension, and fetal growth restriction [35]. • In Norway, most women are admitted to hospital during the labor induction process [36].	• During labor, the fetal heart rate is monitored to identify signs of distress and, thus, intervene if necessary [37]. • There are two approaches to fetal monitoring: intermittent auscultation and continuous electronic monitoring with cardiotocography (CTG) [38]. • Intermittent auscultation is recommended in low-risk deliveries, while CTG is recommended in cases of signs or risk factors for compromised fetal resources [38]. However, CTG is widely used in low-risk deliveries [39, 40].

### Phase 2 identifying and verifying research needs

Research needs were initially identified through literature searches. Subsequently, knowledge users were involved to further identify additional needs, see Fig. 1. The relevance of the research needs identified in the literature were verified through the knowledge user involvement. Likewise, the needs identified by the knowledge users were verified as actual evidence gaps in the literature. An evidence gap was defined as when no up-to-date, relevant, or reliable systematic reviews addressed the question or when up-to-date, relevant, and reliable systematic reviews showed that uncertainty existed [20]. The literature searches and knowledge user involvement are described in greater detail below.

The initial literature searches were conducted by each of the PhD students (KEM and KJA, respectively) during September and October 2019. The search strategies are presented in the Supplementary Files 3 and 4. The searches were conducted for reviews published during the last ten years in the MEDLINE Database and the Cochrane Database of Systematic Reviews, and titles and abstracts were screened for relevance to the scope. In NLR-LISTEN, in which few reviews were retrieved, primary studies were also included. For both NLR-LINO and NLR-LISTEN, relevant papers were read in full text to identify research needs. To ensure comprehensiveness and facilitate prioritization in the next phase of the NLR processes, the topics on the lists of research needs were grouped in a manner that would be meaningful in clinical practice. In both NLR processes, the PhD students repeated the literature searches regularly until February 2020 to identify newly published research needs and ensure that the research needs suggested by the knowledge users represented actual evidence gaps.

The knowledge user involvement in Phase 2 aimed to verify the relevance of the research needs identified from the literature searches and, potentially, add additional research needs. Inspired by the JLA, the initial plan was to arrange workshops for the knowledge user groups in both NLR-LINO and NLR-LISTEN [20]. However, in NLR-LINO, gathering the whole group for a workshop was not feasible within the limited timeframe. Hence, the PhD student held individual one-to-one conversations with the knowledge user group members. Meetings with the women were held in informal settings, while the midwives and obstetrician were met at the hospital during their working hours. All the conversations were face-to-face and lasted approximately one hour, and notes were taken by the PhD student.

In NLR-LISTEN, a face-to-face knowledge user workshop lasting for 1.5 h was arranged. Initially, the project and the objective of the workshop were introduced. The participants then engaged in pairwise discussions on fetal monitoring in low-risk deliveries, guided by specific questions. These pairwise discussions evolved into an open group discussion, with active contributions from the midwives and obstetrician, while the women participated to a lesser extent. The workshop was led by the PhD student and her main supervisor, and notes were taken by a person not involved in the project. After the workshop, the PhD student wrote a summary which was sent to the workshop participants for approval. Following the workshop, the project group discussed concerns regarding the women’s limited level of engagement in the workshop and, thus, their impact as knowledge users in the NLR process. To address this concern and explore whether the women had any additional suggestions for research needs, a separate meeting with the women was arranged. The women expressed a desire to continue participating in the NLR process, emphasizing the importance of including women as knowledge users.

In both NLR-LINO and NLR-LISTEN, any research needs suggested in spontaneous conversations by knowledge users outside the formal knowledge user groups were also noted by the PhD students, for example, during a lunch at the university or at the maternity care units.

### Phase 3 prioritizing research needs

In both projects, prioritizing the research needs included a knowledge user survey, followed by a knowledge user group workshop (see Fig. 1). A survey was considered a feasible approach to collect opinions from various perspectives from all over the country within a short timeframe [41]. The knowledge user surveys were developed by the project groups based on the research needs identified in Phase 2. The research needs presented in the surveys are shown in Table 2 (NLR-LINO) and Table 3 (NLR-LISTEN). Members of the respective knowledge user groups provided written or oral input to ensure that the surveys were understandable and relevant. Two versions of each survey were prepared. The research needs presented were the same but were written in plain language for the women and by using more medical terms for the health professionals. In NLR LINO, the respondents were asked to rate the importance of each research need on a four-point Likert scale, and asked which research need they would prioritize in an open-ended question. The respondents in the NLR-LISTEN survey were asked to select the three needs they considered most important. Both surveys ended with an open-ended question asking, “Is there anything else you would like to add?”. The surveys were anonymous and internet-based. The survey participants were recruited through relevant Facebook groups and at national conferences for midwives and obstetricians. The NLR-LINO project also recruited women at a maternal and child health center. The results from each survey were summarized by the project’s PhD student.

**Table 2:** Number of knowledge users rating each research need as “very important” in the NLR-LINO survey

Research needs*		All *n* = 322 *n* (%)	Women *n* = 173 *n* (%)	Midwives *n* = 105 *n* (%)	Obstetricians *n* = 45 *n* (%)
1	At what gestational week should labor be induced in cases of post term pregnancy?	263 (82)	144 (83)	82 (78)	37 (84)
2	Explore whether and how induction of labor is associated with an increased risk of negative outcomes compared to a spontaneous onset of labor.	241 (75)	112 (65)	91 (87)	38 (86)
3	Compare different methods of labor induction.	221 (69)	111 (64)	76 (72)	34 (77)
4	Explore women’s experiences of induced labor as compared to the spontaneous onset of labor.	220 (68)	112 (65)	88 (84)	20 (46)
5	Explore factors promoting a positive labor experience in induced labor.	220 (68)	125 (72)	72 (69)	23 (52)
6	Establish a scheme to ensure that comprehensive information is provided to women being induced.	209 (65)	118 (68)	68 (65)	23 (52)
7	Test interventions to improve the labor induction process.	198 (62)	107 (62)	63 (60)	28 (64)
8	Develop an approach to categorizing pregnancy risk factors to enable a more individualized and comprehensive assessment of the need to induce labor.	197 (61)	94 (54)	77 (73)	26 (59)
9	Identify predictors of successful/failed labor induction.	192 (60)	84 (49)	70 (67)	38 (86)
10	What progress should be expected in induced labor as compared to labor with spontaneous onset?	164 (51)	74 (43)	57 (54)	33 (75)
11	Examine whether and how labor induction affects breastfeeding after childbirth.	160 (50)	92 (53)	58 (55)	10 (23)
12	Describe differences between institutions and changes over time, including methods and indications for labor induction.	146 (45)	67 (39)	57 (54)	22 (50)
13	Examine why women want/do not want labor induction.	144 (45)	74 (43)	57 (54)	13 (30)
14	Examine whether women in Norway want to be involved in decision-making regarding labor induction.	141 (44)	92 (53)	37 (35)	12 (27)
15	Explore alternative methods of labor induction, such as acupuncture, acupressure, or others.	94 (29)	53 (31)	32 (31)	9 (21)
16	Develop methods for assessing cervical ripeness that are more objective than the Bishop’s score.	94 (29)	56 (32)	30 (29)	8 (18)
17	Examine economic aspects of labor induction.	82 (26)	23 (13)	42 (40)	17 (39)

*Translated from Norwegian

**Table 3:** Number of knowledge users selecting each research need in the NLR-LISTEN survey to be among the three most important

Research need		Total *n* = 466 *n* (%)	Women *n* = 278 *n* (%)	Midwives *n* = 138 *n* (%)	Obstetricians *n* = 50 *n* (%)
1	Whether various organizational factors affect how women are monitored during birth, for example, selection, staffing, training/education, and procedures.	303 (65)	186 (67)	84 (61)	33 (66)
2	Compliance with fetal monitoring guidelines and procedures.	241 (52)	150 (54)	56 (41)	35 (70)
3	Midwives’ preferences, attitudes, and experiences of fetal monitoring in low-risk deliveries.	223 (48)	150 (54)	61 (44)	12 (24)
4	Develop and evaluate a procedure (“framework”) for intermittent auscultation.	150 (32)	75 (27)	55 (41)	20 (40)
5	The women’s preferences and experiences with fetal monitoring in childbirth.	139 (30)	91 (33)	39 (28)	9 (18)
6	Trends in the proportions of low-risk women and uncomplicated births in Norway.	138 (30)	66 (24)	45 (33)	27 (54)
7	Obstetricians’ preferences, attitudes, and experiences regarding fetal monitoring in low-risk deliveries.	109 (23)	61 (22)	36 (26)	12 (24)

Knowledge user group workshops were planned in both NLR processes to discuss the knowledge user survey results and provide recommendations for the research projects. In NLR-LINO, the members of the group were e-mailed the survey results prior to the workshop. The workshop, facilitated and led by the PhD student, was an open group face-to-face discussion lasting for 1.5 h. The PhD student took notes during the workshop and wrote a summary which was sent to the group members for approval. In NLR-LISTEN, the workshop in Phase 3 was cancelled due to the Covid 19 pandemic lockdown.

### **Phase 4 designing the PhD projects**

The final aims and objectives for the PhD projects were based on the results of the previous phases of the NLR processes. In addition, the project groups had to ensure suitability and feasibility within the frame of a PhD project, as well as the competencies and interests of the PhD student and supervisors.

### Ethical issues

Both NLR processes were approved by the Data Protection Services of Sikt – Norwegian Agency for Shared Services in Education and Research, with reference numbers 617,764 and 908,097. We followed the REPRISE Guideline [42] for reporting the priority settings and the GRIPP2 Checklist [43] for reporting the user involvement in our processes (see Supplementary Files 1 and 2 for details).

## Results

**Fig. 2:**
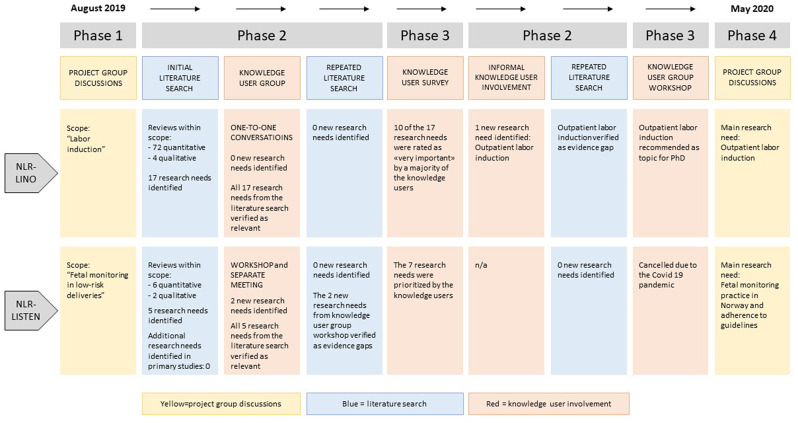
Overview of the results of NLR-LINO and NLR-LISTEN

The main results are presented in Fig. 2, and details are presented for each NLR process separately.

### Phase 1 defining the scope

The preliminary scope of the LINO project was “labor induction,” and this scope remained unchanged after discussions in the project group. In the LISTEN project, the preliminary scope was “fetal monitoring in low-risk deliveries with intermittent auscultation.” Intermittent auscultation involves periodically listening to the fetal heart during labor, in contrast to continuous electronic monitoring [37]. However, during the informal literature review, limited research was found regarding intermittent auscultation. Furthermore, through informal conversations with midwives and obstetricians at the maternity care units, they highlighted that continuous fetal monitoring was also commonly used in clinical practice, even for low-risk deliveries. As a result, the final scope of the NLR-LISTEN project was expanded to “fetal monitoring in low-risk deliveries”, including both intermittent auscultation and continuous fetal monitoring.

### Phase 2 identifying and verifying research needs

In NLR-LINO, the initial literature search for reviews resulted in 120 papers. After 48 papers that were either duplicates, not reviews, or not within the scope were excluded, research needs were identified from 72 quantitative and four qualitative reviews. The findings were grouped into 17 research needs, as presented in Table 2. No new research needs were identified in the subsequent literature searches. The one-to-one conversations with the members of the knowledge user group did not reveal additional research needs beyond the 17 needs identified through the literature search. The knowledge user group members verified all 17 research needs as relevant, with adjustments for Norwegian settings. For example, need number 1 in Table 2 was modified to center on post-term pregnancies, reflecting the current debate in Norway. After initiating the subsequent phase of the NLR process, “Phase 3; Prioritizing research needs”, clinicians at the maternity care unit suggested a new research need during a spontaneous conversation: outpatient treatment of labor induction. Extensive discussions within the Norwegian maternity care community were prompted by the publication of a Danish paper [44]. This paper introduced outpatient care as a potential alternative for medical labor induction, expanding the options available to women. Although the need was identified too late to be included in the knowledge user survey conducted during Phase 3, the project group added “outpatient labor induction” as a potential research need in the NLR process.

In NLR-LISTEN, the initial literature search for reviews yielded 27 papers, with 19 reviews being excluded due to their focus on high-risk deliveries. The full texts of the remaining six quantitative and two qualitative reviews were read, and five distinct research needs were identified. The additional search for primary studies resulted in 160 papers. However, all of them either fell outside the scope of NLR-LISTEN, were already included in the reviews, or covered by already identified research needs. During the knowledge user group workshop, a substantial part of the conversation revolved around the midwives’ experiences of many women not meeting the criteria for intermittent auscultation, staffing shortages, and personnel’s lack of experience using intermittent auscultation. This led to the identification of two additional research needs; see research needs numbers one and six in Table 3. The conversation occasionally touched upon high-risk pregnancies, but these topics were not carried forward, as they fell beyond the scope of the process. All research needs from the literature search were verified as relevant. A subsequent literature search verified that the research needs identified during the workshop were actual evidence gaps. No new research needs were identified through the repeated literature searches.

### Phase 3 prioritizing research needs

A total of 322 women and health professionals responded to the NLR-LINO knowledge user survey (see Table 2). Most respondents rated 10 of the 17 research needs as “very important.” Although respondents were also invited to suggest additional research needs, no new ones that fell within the scope were identified. During the knowledge user workshop, all group members actively contributed and commented on one another’s observations and thoughts. They agreed that the comments in response to the open-ended questions were aligned with the ratings of the research needs. There were no strong opinions regarding the prioritization of research needs. During the workshop, the PhD student presented outpatient labor induction as a potential main research need, as suggested by the clinicians late in the process. The knowledge user group recognized this need as relevant and in line with the results of the knowledge user survey, addressing several highly prioritized research needs, particularly needs 5 and 7 in Table 2. Thus, the knowledge user group recommended outpatient labor induction as the main research need for the LINO project. The group provided valuable inputs into the study design and feasibility, highlighting important considerations for the women participating in the study and the importance of including women’s views. For example, the women emphasized the value of midwives reaching out to them for telephone consultations during home-based treatment, as it made them feel better cared for compared to if they had to contact the hospital themselves.

A total of 466 women and health professionals responded to the NLR-LISTEN knowledge user survey (see Table 3). 65% of the knowledge users prioritized one of the needs identified in the knowledge user group workshop as most important. No research needs within the scope were suggested through the survey that had not already been identified. In the open-ended questions, a recurring theme related to several of the research needs was mentioned: many, especially midwives, experienced that continuous fetal monitoring was being used in low-risk deliveries, despite the guidelines recommending the use of intermittent auscultation. The NLR-LISTEN knowledge user group workshop in Phase 3, originally scheduled for March 2020, was canceled because of the COVID-19 pandemic lockdown. The project group deemed it inappropriate to prioritize the workshop given the widespread impact of the lockdown on society in general, as well as the substantial additional demands on healthcare personnel in particular. As the deadline for the NLR process was in May 2020, it was not feasible to postpone the workshop for a later date. Consequently, the project group relied solely on the survey feedback to prioritize research needs for the PhD project.

### Phase 4 designing the PhD projects

The NLR-LINO project group defined “outpatient labor induction” as the main research need for the PhD project, as recommended by the knowledge user group. Although it was not explicitly mentioned in the knowledge user survey, there was a significant interest and enthusiasm regarding this need within the maternity care community following the recent publication of a Danish paper [44]. Additionally, outpatient labor induction aligned with the survey results, as it covered several of the needs rated as “very important” by most of the respondents (see Table 4). The survey results and input from the knowledge user group were essential in the design of the PhD project. Consequently, the project group chose objectives focusing on clinically relevant outcomes and women’s experiences. Telephone consultations, as suggested by the women in the knowledge user group, were incorporated into the study protocol, and a day-to-day diary was implemented to capture the participating women’s experiences. Moreover, the study design was tailored to fit existing clinical routines rather than adjusting the clinical practice for the purpose of the study.

**Table 4:** The final objectives of the PhD projects

	LINO	LISTEN
**Scope**	Labor induction	Fetal monitoring in low-risk deliveries
**Research need**	Main research need: outpatient labor induction. Included aspects of Needs 4, 5, 6, 7, 9, 13, and 14 in Table 2.	Main research need: fetal monitoring practice in Norway and adherence to fetal monitoring guidelines. Included aspects of Needs 1, 2, 3, 6, and 7 in Table 3.
**Aim**	To investigate whether outpatient induction of labor is beneficial in a Norwegian setting.	To investigate the methods used for fetal monitoring in low-risk deliveries in Norway and evaluate adherence to evidence-based practice while also exploring potential reasons for any deviations.
**Objectives**	Objective 1: Investigate the feasibility and clinical outcomes of inducing in an outpatient setting as compared to an inpatient setting in Norway. Objective 2: Explore low-risk nulliparous women’s experiences of labor induction in inpatient and outpatient settings.	Objective 1: Which methods are used for fetal monitoring in Norway; how many deliveries are low-risk, and how many are suitable for monitoring with intermittent auscultation? Objective 2: What are barriers and facilitators to use intermittent auscultation when monitoring low-risk deliveries? Objective 3: What are determinants that positively or negatively influence midwives’ and obstetricians’ adherence to fetal monitoring guidelines?

The NLR-LISTEN project group defined “fetal monitoring practice in Norway and adherence to fetal monitoring guidelines” as the main research need for the PhD project. A recurring theme throughout the NLR-LISTEN process, both in the knowledge user group workshop and the survey, was the extent to which evidence-based practices are used in low-risk deliveries and the best ways to promote and encourage evidence-based practice. The project group designed three studies based on the three highest-rated research needs in the knowledge user survey. Additionally, needs 6 and 7 were considered relevant in the context of evidence-based practice in fetal monitoring. Consequently, the final PhD project covered five of the seven research needs found in the survey (see Table 4). Due to the cancelation of the knowledge user group workshop in Phase 3, the knowledge user group did not influence the design of the final PhD project.

## Discussion

In this paper, we have described the implementation of NLR processes for identifying, verifying, and prioritizing research needs in two different PhD projects. Through literature searches and knowledge user involvement, we have ensured that the objectives of the PhD projects address actual evidence gaps that are relevant to the users of the knowledge.

The NLR-LINO and NLR-LISTEN processes shared many similarities as part of the Bridgebuilder Initiative, beginning at the same time and having similar timeframes, budgets, and resources. Both projects addressed the field of maternity care, and both PhD students were midwives. However, although these processes are described together in this paper due to their similarities, there are also differences worth mentioning. The first key distinction between the two NLR processes lies in their scopes, as defined in Phase 1. In NLR-LINO, with its broader scope of “labor induction,” as many as 76 reviews were identified, revealing evidence gaps that were grouped into 17 research needs. In contrast, NLR-LISTEN, with its narrower scope of “fetal monitoring in low-risk deliveries”, identified only eight reviews and seven research needs. Consequently, the NLR-LINO knowledge user survey was more extensive, asking the respondents to rate the importance of each of the 17 research needs, without prioritizing them. Meanwhile, the NLR-LISTEN survey was more concise, requiring the participants to select the three needs they considered most important. Hence, the NLR-LISTEN approach allowed for a more explicit prioritization of research needs by the knowledge user, while the NLR-LINO approach distinguished between relevant and less relevant needs.

A second contrast between the NLR processes relates to the selection of research needs for the PhD projects. When the LISTEN project was designed, the three highest-rated needs from the knowledge user survey were incorporated. These needs were also consistent with the discussions in the knowledge user group workshop that were carried out in Phase 2 of the process, and the comments received from the open-ended question in the survey. In contrast, the LINO was designed based on one main research need that was identified late in the NLR process: outpatient labor induction. Hence, this research need was not part of the user survey. Instead, the inclusion of the need was based on a significant interest and enthusiasm demonstrated by the maternity care providers, following the publication and discussion of a study [44]. This interest led the project group to feel confident in the relevance of the research need to all knowledge users, particularly considering the potential for expanding pregnant women’s options. Hence, the project group concluded that the purpose of the NLR process, to address actual evidence gaps relevant to knowledge users [22], was fulfilled, even without incorporating the top three rated research needs from the survey. This view was supported by all members of the knowledge user group.

A third distinct difference between the NLR processes was the level of participation among the women in the knowledge user group workshops. Both knowledge user groups consisted of members with comparable backgrounds and levels of expertise. In NLR-LINO, the women were actively involved, reaffirming their significant role as group members. In contrast, the women in the NLR-LISTEN group were less active during the workshop discussions. A possible explanation for the varying engagement levels could be the fact that NLR-LISTEN’s scope was more technically oriented and, thus, the workshop was largely driven by the health professionals’ perspectives. However, during the separate meeting with the women, they emphasized their interest in the process and the importance of their participation. The substantial response from women on the NLR-LISTEN knowledge user survey reinforced this view.

A final distinction between the NLR processes relates to the knowledge user groups’ influence on the final objectives of the PhD project. In NLR-LISTEN, the cancellation of the Phase-3 workshop limited the direct input from the knowledge user group, while the NLR-LINO knowledge user group had a substantial impact on the final objectives and project design. The active involvement in NLR-LINO may have strengthened the knowledge users’ sense of ownership of the project, potentially increasing their motivation to pursue additional involvement in the PhD project.

In both NLR processes, a challenge arose concerning the representativeness of knowledge users, both within the knowledge user groups and in the knowledge user surveys. Both knowledge user groups were comprised of highly educated members of the majority population, resulting in a socio-economic and cultural bias [6]. Researchers often involve user organizations to ensure a broader range of perspectives [45]; however, few such organizations existed in maternal health at the time. Thus, three of the four women in the knowledge user groups were recruited via informal channels. One advantage of this approach was that the absence of organizational ties may have allowed the women to express their thoughts freely [42]. Moreover, all maternity care providers were recruited from the same hospital, potentially limiting the diversity of professional perspectives. Regarding the knowledge user surveys, participants were primarily recruited through social media, with maternity care providers also being recruited at a national conference. This approach facilitated geographical diversity, whereas the diversity of socio-economic groups was unknown. Additionally, the surveys were available only in Norwegian, excluding individuals who were not fluent in the language. Although efforts were made to recruit women for the NLR-LINO survey at a maternal and child health center in a low socio-economic area, this was time consuming and resulted in few additional participants. These recruitment biases prompted both the knowledge user- and project groups to actively consider the perspectives of potentially underrepresented groups and be cautious in generalizing the results of the knowledge user survey.

Although NLR is inspired by the JLA, our processes were notably smaller and constrained by time and resources [20, 22]. While the aim of a JLA Priority Setting Partnership is to create a Top 10 list of research areas within a scope, our aim was to ensure that we addressed actual and relevant research needs in our PhD projects [20, 22]. Hence, we argue that an NLR process does not require the same level of systematics or rigor as a JLA process. To strike a balance between a meaningful process and avoiding wasteful resource overuse, we made pragmatic choices and assessments of the appropriate level of resource allocation for each NLR activity. Consequently, the literature searches were conducted individually by the PhD students, without a thorough systematic approach, potentially resulting in overlooking evidence gaps. However, the expertise within the project- and knowledge user groups provided confidence that essential research needs were not being neglected. In addition, the knowledge user group workshops did not involve extensive data collection or systematic and documented qualitative analysis. Nonetheless, summaries of the workshops were shared with the knowledge user group members for validation. Moreover, there was an imbalance in the NLR-LISTEN knowledge user group, with two women and five clinicians. This was due to difficulties in recruiting women within the limited timeframe. Recognizing midwives as the primary users and interpreters of intermittent auscultation, their participation was considered most crucial for the project’s scope. Hence, the participation of two women was considered sufficient for the process. Finally, the knowledge user surveys lacked consistency and testing for validity and reliability. There was a significant variation in the phrasing of the research needs, especially in the NLR-LINO survey, but the potential impact of this variation is unknown. However, the survey questions were reviewed by the knowledge user group members in advance to ensure clarity. Additionally, the inclusion of the open-ended question provided an opportunity for the respondents to suggest additional research topics.

To the best of our knowledge, no research priority settings have been published regarding labor induction or fetal monitoring [18, 25]. In 2019, Graham et al. conducted a systematic review of research priorities in the field of women’s health [25]. They identified 12 studies, seven of which were related to pregnancy and childbirth, but none were specifically related to labor induction or fetal monitoring. All studies included in their review used the JLA method. In 2023, Mossinger et al. published a systematic review on research priorities within maternal and perinatal health [18]. They identified 62 research prioritization projects, none of which were related to labor induction or fetal monitoring. The most commonly employed methods for prioritization were Delphi, the Child Health Nutrition Research Initiative (CHNRI) and JLA. They reported that only 34% of the projects included a literature search to verify the suggested research needs as actual evidence gaps. NLR-LINO and NLR-LISTEN were based on the same principles [22] as the NLR-processes described by Madsen et al. [29], Slåtsveen et al. [30] and Solbakken et al. [31], all fellow PhD students in the Bridgebuilder Initiative. The topics of their NLR processes included the everyday life of older patients with multimorbidity [29], the trust model in community home-based health care services [30], and transitional care for patients with acute stroke [31]. While the NLR principles remained the same, there was significant variation in how the processes were implemented in terms of the extent and role of literature searches, knowledge user groups, and the use of knowledge user surveys. For example, they primarily identified research needs through user group surveys, while planning to prioritize the needs in knowledge user workshops (although cancelled due to the Covid 19 pandemic lockdown). In contrast to our processes, they also included caregivers as knowledge users, in addition to service users and clinicians [29–31].

Incorporating NLR as part of a PhD project represents a novel approach, raising the question of its advisability for future projects. In this paper, we have demonstrated that NLR emerges as a viable approach to enhance knowledge user involvement in prioritizing research, even within the context of PhD projects. However, debates may arise regarding the allocation of time and resources. If additional time required for an NLR process is not added to a given PhD project, the time available for conducting the research is reduced. On the other hand, as the objectives for research following an NLR process are answering relevant questions, it is conceivable that the knowledge generated could be implemented more rapidly in practice. In NLR-LINO and NLR-LISTEN, the processes provided the PhD students with valuable competencies early in their academic careers, potentially influencing their future choices and opportunities. They provided hands-on experience in understanding how research can address research needs in society and fostered discussions on the boundaries of what constitutes research. In addition, the PhD students’ experience with knowledge user involvement methodologies may lower the barriers to incorporating similar approaches in their future projects. Finally, the NLR process provided the PhD students with ownership of their projects and the entire research process. Our recommendations for incorporating NLR in PhD projects include developing a protocol to guide the process while remaining open to pragmatic choices along the way. It is important to ensure the presence of a dedicated project group with prior experience with similar approaches and to allocate additional time and training beyond standard PhD projects.

## Conclusions

The NLR processes described in this paper ensured PhD projects addressing research needs that are identified, verified, and prioritized through knowledge user involvement and literature reviews. Although the process did not generate extensive, generalizable, or prioritized lists of all potential research needs within the scopes, we are confident that the objectives of the PhD projects cover actual evidence gaps that are relevant to knowledge users. The findings showed how the initial scope impacted the subsequent phases of the process, as well as the importance of pragmatic decision-making during the entire process and balancing between a meaningful process and resource utilization. Despite its benefits, it is essential to recognize that NLR requires dedicated resources, and if it is integrated into PhD projects, additional time and training should be allocated accordingly.

## Electronic supplementary material

Below is the link to the electronic supplementary material.

Supplementary Material 1

Supplementary Material 2

Supplementary Material 3

Supplementary Material 4

## Data Availability

No datasets were generated or analysed during the current study.

## Abbreviations

NLR: Needs Led Research
LINO: Labor induction inpatient and outpatient
NLR-LINO: The Needs Led Research process in the LINO project
LISTEN: Low risk intrapartum fetal monitoring
NLR-LISTEN: The Needs Led Research process in the LISTEN project
JLA: James Lind Alliance

## References

[1] Chalmers I, Glasziou P. Avoidable waste in the production and reporting of research evidence. Obstet Gynecol. 2009;114(6):1341–5. 19935040 10.1097/AOG.0b013e3181c3020d

[2] Series from the Lancet journals. Research: increasing value, reducing waste. Lancet. 2014. https://www.thelancet.com/series/research

[3] Chalmers I, Bracken MB, Djulbegovic B, Garattini S, Grant J, Gülmezoglu AM, et al. How to increase value and reduce waste when research priorities are set. Lancet. 2014;383(9912):156–65. 24411644 10.1016/S0140-6736(13)62229-1

[4] Crowe S, Fenton M, Hall M, Cowan K, Chalmers I. Patients’, clinicians’ and the research communities’ priorities for treatment research: there is an important mismatch. Res Involv Engagem. 2015;1:2. 29062491 10.1186/s40900-015-0003-x PMC5598091

[5] Tallon D, Chard J, Dieppe P. Relation between agendas of the research community and the research consumer. Lancet. 2000;355(9220):2037–40. 10885355 10.1016/S0140-6736(00)02351-5

[6] Viergever R, Olifson S, Ghaffar A, Terry R. A checklist for health research priority setting: nine common themes of good practice. Health Res Policy Syst. 2010;8(1):36. 21159163 10.1186/1478-4505-8-36 PMC3018439

[7] Young C, Horton R. Putting clinical trials into context. Lancet. 2005;366(9480):107–8. 16005318 10.1016/S0140-6736(05)66846-8

[8] Lund H, Juhl CB, Norgaard B, Draborg E, Henriksen M, Andreasen J, et al. Evidence-based Research Series-Paper 2: using an evidence-based Research approach before a new study is conducted to ensure value. J Clin Epidemiol. 2021;129:158–66. 32987159 10.1016/j.jclinepi.2020.07.019

[9] Habre C, Tramer MR, Popping DM, Elia N. Ability of a meta-analysis to prevent redundant research: systematic review of studies on pain from propofol injection. BMJ. 2014;348:g5219. 25161280 10.1136/bmj.g5219 PMC4145062

[10] Garritty C, Tricco AC, Smith M, Pollock D, Kamel C, King VJ. Rapid Reviews methods Series: involving patient and public partners, healthcare providers and policymakers as knowledge users. BMJ Evidence-based Med. 2024;29(1):55–61. 10.1136/bmjebm-2022-112070 PMC10850627 37076265

[11] Canadian Institutes of Health Research: Knowledge User Engagement. 2016. https://www.cihr-irsc.gc.ca/e/49505.html. Accessed 27 Feb 2024.

[12] Grill C. Involving stakeholders in research priority setting: a scoping review. Res Involv Engagem. 2021;7(1):75. 34715932 10.1186/s40900-021-00318-6 PMC8555197

[13] Norwegian Ministry of Health and Care Services. HelseOmsorg21. Et kunnskapssystem for bedre folkehelse. Nasjonal forskning- og innovasjonsstrategi for helse og omsorg. [HealthCare21: A Knowledge System for Improved Public Health. National Research and Innovation Strategy for Health and Care.] 2014. https://www.regjeringen.no/contentassets/8ab2fd5c4c7746dfb51e3f64cd4d71aa/helseomsorg21_strategi_web.pdf?id=2266705. Accessed 11 Jan 2024.

[14] Norwegian Ministry of Health and Care Services. Meld. St. 16. (2010–2011) Report to the Storting (white paper) summary: National Health and Care services Plan (2011-2015). 2011.https://www.regjeringen.no/en/dokumenter/meld.-st.-16-2010-2011/id639794/2011. Accessed 11 Jan 2024.

[15] Lund H, Tang L, Poulsen I, la Cour K, Bjerrum M, Nielsen CV, Maribo T. Lack of systematicity in research prioritisation processes - a scoping review of evidence syntheses. Syst Reviews. 2022;11(1):277. 10.1186/s13643-022-02149-2 PMC9784020 36564846

[16] Wong EC, Maher AR, Motala A, Ross R, Akinniranye O, Larkin J, Hempel S. Methods for Identifying Health Research Gaps, needs, and priorities: a scoping review. J Gen Intern Med. 2022;37(1):198–205. 34748098 10.1007/s11606-021-07064-1 PMC8738821

[17] Nyanchoka L, Tudur-Smith C, Thu VN, Iversen V, Tricco AC, Porcher R. A scoping review describes methods used to identify, prioritize and display gaps in health research. J Clin Epidemiol. 2019;109:99–110. 30708176 10.1016/j.jclinepi.2019.01.005

[18] Mossinger C, Manerkar K, Crowther CA, Harding JE, Groom KM. Research priorities for maternal and perinatal health clinical trials and methods used to identify them: a systematic review. Eur J Obstet Gynecol Reprod Biol. 2023;280:120–31. 36455392 10.1016/j.ejogrb.2022.11.022

[19] Manafò E, Petermann L, Vandall-Walker V, Mason-Lai P. Patient and public engagement in priority setting: a systematic rapid review of the literature. PLoS ONE. 2018;13(3):e0193579. 29499043 10.1371/journal.pone.0193579 PMC5834195

[20] Cowan K, Oliver S. The James Lind Alliance Guidebook, Version 10: National Institute for Health and Care Research. 2021. http://www.jla.nihr.ac.uk/jla-guidebook/. Accessed 15 Jan 2024.

[21] Greenhalgh T, Hinton L, Finlay T, Macfarlane A, Fahy N, Clyde B, Chant A. Frameworks for supporting patient and public involvement in research: systematic review and co-design pilot. Health Expect. 2019;22(4):785–801. 31012259 10.1111/hex.12888 PMC6737756

[22] Ormstad H, Jamtvedt G, Svege I, Crowe S. The Bridge Building Model: connecting evidence-based practice, evidence-based research, public involvement and needs led research. Res Involv Engagem. 2021;7(1):77. 34717755 10.1186/s40900-021-00320-y PMC8557598

[23] Downe S, Finlayson K, Oladapo OT, Bonet M, Gulmezoglu AM. What matters to women during childbirth: a systematic qualitative review. PLoS ONE. 2018;13(4):e0194906. 29664907 10.1371/journal.pone.0194906 PMC5903648

[24] World Health Organization. WHO recommendations: intrapartum care for a positive childbirth experience. 2018. https://www.who.int/publications/i/item/9789241550215 30070803

[25] Graham L, Illingworth B, Showell M, Vercoe M, Crosbie EJ, Gingel LJ, et al. Research priority setting in women’s health: a systematic review. BJOG: Int J Obstet Gynecol. 2020;127(6):694–700. 10.1111/1471-0528.16150 32011073

[26] Tomlinson J, Medlinskiene K, Cheong VL, Khan S, Fylan B. Patient and public involvement in designing and conducting doctoral research: the whys and the hows. Res Involv Engagem. 2019;5:23. 31428458 10.1186/s40900-019-0155-1 PMC6697942

[27] Dawson S, Ruddock A, Parmar V, Morris R, Cheraghi-Sohi S, Giles S, Campbell S. Patient and public involvement in doctoral research: reflections and experiences of the PPI contributors and researcher. Res Involv Engagem. 2020;6:23. 32426162 10.1186/s40900-020-00201-w PMC7216324

[28] Oslo Metropolitan University - Faculty of Health Sciences. Bridge-Building Initiative. 2021. https://www.oslomet.no/en/about/hv/research/bridge-building-initiative. Accessed 10 Nov 2023.

[29] Madsen K, Wibe T, Bye A, Debesay J, Bergland A. Top 10 research priorities to improve the everyday life of older patients with multimorbidity: a James Lind Alliance (JLA) inspired Priority setting Partnership (PSP). Tidsskrift Omsorgsforskning. 2021;7(2):57–68. 10.18261/issn.2387-5984-2021-02-05

[30] Slåtsveen R-E, Wibe T, Halvorsrud L, Lund A. Needs-led research: a way of employing user involvement when devising research questions on the trust model in community home-based health care services in Norway. Res Involv Engagem. 2021;7(1):43. 34158122 10.1186/s40900-021-00291-0 PMC8218277

[31] Solbakken LM, Langhammer B, Sundseth A, Brovold T. Transitional care for patients with acute stroke-A priority-setting project. Health Expect. 2022;25(4):1741–52. 35501973 10.1111/hex.13517 PMC9327821

[32] Smits D-W, van Meeteren K, Klem M, Alsem M, Ketelaar M. Designing a tool to support patient and public involvement in research projects: the involvement matrix. Res Involv Engagem. 2020;6:30. 32550002 10.1186/s40900-020-00188-4 PMC7296703

[33] Landsforeningen 1001 dager [The National Organization for the First. 1001 Days]. https://www.landsforeningen1001dager.no. Accessed 31 Jul 2024.

[34] The Norwegian Institute of Public Health. Medisinsk fødselsregister - statistikkbank, F8 Fødselsstart og induksjon [Medical Birth Registry of Norway - F8 Labor onset and labor induction]. http://statistikkbank.fhi.no/mfr/. Accessed 02 Feb 2024.

[35] Sørbye IK, Oppegaard KS, Weeks A, Marsdal K, Jacobsen AF. Induction of labor and nulliparity: a nationwide clinical practice pilot evaluation. Acta Obstet Gynecol Scand. 2020;99(12):1700–9. 32609877 10.1111/aogs.13948

[36] Oppegård KS, Dögl M, Sun C, Hill S, Ween-Velken M, Sørbye IK. Induksjon/igangsetting av fødsel - Modning av cervix/livmorhalsen før fødsel [Labor induction - cervical ripening]. Norwegian Gynecological Association. 2020. ISBN 978-82-692382-0-4. https://www.legeforeningen.no/foreningsledd/fagmed/norsk-gynekologisk-forening/veiledere/veileder-i-fodselshjelp/induksjonigangsettelse-av-fodsel-modning-av-cervixlivmorhalsen-for-fodsel/. Accessed 26 Jan 2022.

[37] Devane D, Lalor JG, Daly S, McGuire W, Cuthbert A, Smith V. Cardiotocography versus intermittent auscultation of fetal heart on admission to labour ward for assessment of fetal wellbeing. Cochrane Database Syst Rev. 2017;1:CD005122. 28125772 10.1002/14651858.CD005122.pub5 PMC6464914

[38] Kessler J, Blix E, Jettestad M, Myklestad K, Nygaard B, Nistov L et al. Fosterovervåkning under fødsel, avnavling og syre-baseprøver fra navlesnor [Intrapartum fetal monitoring]. Norwegian Gynecological Association [Internet]. 2022 November 5, 2023]; ISBN 978-82-692382-0-4. https://www.legeforeningen.no/foreningsledd/fagmed/norsk-gynekologisk-forening/veiledere/veileder-i-fodselshjelp/fosterovervakning-under-fodsel-avnavling-og-syre-baseprover-fra-navlesnor

[39] Jørandli K, Nese A, Vik E, Aasekjær K. Use of admission CTG in low-risk parous women: a clinical audit. Sykepleien Forskning. 2019; 14(78661). https://sykepleien.no/en/forskning/2020/03/use-admission-ctg-low-risk-parous-women-clinical-audit

[40] Rosset IK, Lindahl K, Blix E, Kaasen A. Recommendations for intrapartum fetal monitoring are not followed in low-risk women: a study from two Norwegian birth units. Sex Reproductive Healthc. 2020;26:100552. 10.1016/j.srhc.2020.100552 33038758

[41] Bryant J, Sanson-Fisher R, Walsh J, Stewart J. Health research priority setting in selected high income countries: a narrative review of methods used and recommendations for future practice. Cost Eff Resource Allocation. 2014;12:23. 10.1186/1478-7547-12-23 PMC4396165 25873787

[42] Tong A, Synnot A, Crowe S, Hill S, Matus A, Scholes-Robertson N, et al. Reporting guideline for priority setting of health research (REPRISE). BMC Med Res Methodol. 2019;19(1):243. 31883517 10.1186/s12874-019-0889-3 PMC6935471

[43] Staniszewska S, Brett J, Simera I, Seers K, Mockford C, Goodlad S, et al. GRIPP2 reporting checklists: tools to improve reporting of patient and public involvement in research. Res Involv Engagem. 2017;3:13. 29062538 10.1186/s40900-017-0062-2 PMC5611595

[44] Helmig RB, Hvidman LE. An audit of oral administration of Angusta R (misoprostol) 25 micro g for induction of labor in 976 consecutive women with a singleton pregnancy in a university hospital in Denmark. Acta Obstet Gynecol Scand. 2020;99(10):1396–402. 32311758 10.1111/aogs.13876

[45] Agyei-Manu E, Atkins N, Lee B, Rostron J, Dozier M, Smith M, McQuillan R. The benefits, challenges, and best practice for patient and public involvement in evidence synthesis: a systematic review and thematic synthesis. Health Expect. 2023;26(4):1436–52. 37260191 10.1111/hex.13787 PMC10349234

