# Supplementary material for: Needs led research: ensuring relevant research in two PhD projects within maternity care
Source: Res Involv Engagem. 2024 Sep 12;10:95. doi: 10.1186/s40900-024-00627-6 (PMC11391717; doi:10.1186/s40900-024-00627-6)
Supplement: Supplementary file 1 — Supplementary Material 1 [file 40900_2024_627_MOESM1_ESM.pdf]

Additional File 1. REPRISE guideline (1)

No	Item	Descriptor and/or *examples*	
**A**	**Context and scope**		
1	Define geographical scope	Global, regional, national, city, local area, institutional/organisational level, health service	Yes
2	Define health area, field, focus	Disease or condition specific, interventions, healthcare delivery, health system	Yes
3	Define end-users of research	Intended beneficiaries e.g. general population or a specific population based on demographic (age, gender), clinical (disease, condition), or other characteristics	Yes
4	Define the target audience of the priorities	Policy makers, funders, researchers, industry	Yes
5	Identify the broad research area	Public health, health services research, clinical research, basic science	Yes
6	Identify the type of research question	Ethology, diagnosis, prevention, treatment (interventions), prognosis, health services, psychosocial, behavioural and social science, economic evaluation, implementation	N/A
7	Define the time frame	Interim, short-term, long-term priorities, plans to revise and update	Yes
**B**	**Governance and team**		
8	Describe selection of the leadership and management team	Those responsible for initiating, developing, and guiding the process for priority setting e.g. Steering Committee, Advisory Group, Technical Experts	Yes
9	Describe the characteristics of the team, and the networks they represent	Stakeholder groups, organisations or networks represented, characteristics (demographics, experience, expertise)	Yes
10	Describe any training or experience in priority setting	Consultants or advisors with experience in priority setting	Yes
**C**	**Framework for priority setting**		
11	State the framework used (if any)	James Lind Alliance, COHRED, CHNRI, no framework	Yes
**D**	**Stakeholders or participants**		
12	Define the inclusion criteria for stakeholders involved in priority-setting	Patients, caregivers, general community, health professionals, researchers, policy makers, non-governmental organisations, government, industry; specific groups including vulnerable and marginalized populations	Yes
13	State the strategy or method for identifying and engaging stakeholders	Partnership with organizations, social media, recruitment through hospitals	Yes
14	Indicate the number of participants and/or organisations involved	Number of individuals and organisations, include number by stakeholder group	Yes
15	Describe the characteristics of stakeholders	Stakeholder group, demographic characteristics, areas of interest and expertise, discipline, affiliations	Yes
16	State if reimbursement for participation was provided	Cash, vouchers, certificates, acknowledgement; what purpose e.g. travel, accommodation, honorarium	Yes
**E**	**Identification and collection of research priorities**		
17	Describe methods for collecting priorities from stakeholders	Methods e.g. Delphi survey, surveys, nominal group technique, interviews, focus groups, meetings, workshops; prioritization e.g. voting, ranking; mode e.g. face-to-face, online; may be informed by evidence e.g, systematic reviews, reviews of guidelines/other documents	Yes
18	Describe methods for collating and categorizing priorities	Taxonomy or other framework used to organise, summarise, and aggregate topics or questions	Yes
19	Describe methods and reasons for removing priorities	Based on scope, clarity, definition, duplication, other criteria	Yes
20	Describe methods for refining or translating priorities into research topics or questions	Reviewed by Steering Committee or project team	Yes

21	Describe methods for checking whether research questions or topics have been answered	Systematic reviews, evidence mapping, consultation with experts	Yes
22	Describe number of research questions or topics	Number of priorities at each stage of the process	Yes
**F**	**Prioritisation of research topics/questions**		
23	Describe methods and criteria for prioritising research topics or questions	Methods e.g. Delphi survey, surveys, nominal group technique, interviews, focus groups, meetings, workshops; prioritisation e.g. voting, ranking; mode e.g. face-to-face, online; criteria e.g. need, feasibility, novelty, equity	Yes
24	Provide reasons for excluding research topics/questions	Thresholds for ranking scores, proportions, votes; other criteria	Yes
**G**	**Output**		
25	Specificity of research priorities are clear	Area, topic, questions, PICO (population, intervention, comparator, outcome)	Yes
**H**	**Evaluation and feedback**		
26	Describe how the process of prioritization was evaluated	Survey, workshop	Yes
27	Describe the approach for feeding back priorities to stakeholders and/or to the public; and how feedback was addressed and integrated	Public meetings or workshop, newsletters, website, email	N/A
**I**	**Implementation**		
28	Outline the strategy or action plans for implementing priorities	Communication with target audience, via policies and funding	Yes
29	Describe evaluation of impact	Integration in decision-making, funding allocation	Yes
**J**	**Funding and conflict of interest**		
30	State sources of funding	Name sources of funding for the priority-setting exercise	Yes
31	Outline the budget and/or cost	Indicate budget and cost	Yes
32	Provide declaration of conflict of interest	Statement of conflict of interest	Yes

Reference

1. Tong A, Synnot A, Crowe S, Hill S, Matus A, Scholes-Robertson N, et al. Reporting guideline for priority setting of health research (REPRISE). BMC Medical Research Methodology. 2019;19(1):243.
