# Supplementary material for: Needs led research: ensuring relevant research in two PhD projects within maternity care
Source: Res Involv Engagem. 2024 Sep 12;10:95. doi: 10.1186/s40900-024-00627-6 (PMC11391717; doi:10.1186/s40900-024-00627-6)
Supplement: Supplementary file 2 — Supplementary Material 2 [file 40900_2024_627_MOESM2_ESM.pdf]

Additional File 1 – GRIPP 2, Long form (1)

Section and topic	Item	Reported on page No
**Section 1: Abstract of paper**		
1a: Aim	Report the aim of the study	2
1b: Methods	Describe the methods used by which patients and the public were involved	2
1c: Results	Report the impacts and outcomes of PPI in the study	2
1d: Conclusions	Summarize the main conclusions of the study	2
1e: Keywords	Include PPI, "patient and public involvement," or alternative terms as keywords	2
**Section 2: Background to paper**		
2a: Definition	Report the definition of PPI used in the study and how it links to comparable studies	4-5
2b: Theoretical Underpinnings	Report the theoretical rationale and any theoretical influences relating to PPI in the study	3-5
2c: Concepts and theory development	Report any conceptual or theoretical models, or influences, used in the study	4
**Section 3: Aims of paper**		
3: Aim	Report the aim of the study	5
**Section 4: Methods of paper**		
4a: Design	Provide a clear description of methods by which patients and the public were involved	6-11
4b: People involved	Provide a description of patients, carers, and the public involved with the PPI activity in the study	7
4c: Stages of involvement	Report on how PPI is used at different stages of the study	6-11
4d: Level or nature of Involvement	Report the level or nature of PPI used at various stages of the study	6-11

Section and topic	Item	Reported on page No
**Section 5: Capture or measurement of PPI impact**		
5a: Qualitative evidence of impact	If applicable, report the methods used to qualitatively explore the impact of PPI in the study	Not applicable
5b: Quantitative evidence of impact	If applicable, report the methods used to quantitatively measure or assess the impact of PPI	Not applicable
5c: Robustness of Measure	If applicable, report the rigour of the method used to capture or measure the impact of PPI	18-21
**Section 6: Economic assessment**		
6: Economic assessment	If applicable, report the method used for an economic assessment of PPI	Not applicable
**Section 7: Study results**		
7a: Outcomes of PPI	Report the results of PPI in the study, including both positive and negative outcomes	12-17
7b: Impacts of PPI	Report the positive and negative impacts that PPI has had on the research, the individuals involved (including patients and researchers), and wider impacts	12-17
7c: Context of PPI	Report the influence of any contextual factors that enabled or hindered the process or impact of PPI	12-17
7d: Process of PPI	Report the influence of any process factors, that enabled or hindered the impact of PPI	12-17
7ei: Theory development	Report any conceptual or theoretical development in PPI that have emerged	Not applicable
7eii: Theory development	Report evaluation of theoretical models, if any	Not applicable
7f: Measurement	If applicable, report all aspects of instrument development and testing (eg, validity, reliability, feasibility, acceptability, responsiveness, interpretability, appropriateness, precision)	Not applicable
7 g: Economic assessment	Report any information on the costs or benefit of PPI	Not applicable
**Section 8: Discussion and conclusions**		

Section and topic	Item	Reported on page No
8a: Outcomes	Comment on how PPI influenced the study overall. Describe positive and negative effects	19-23
8b: Impacts	Comment on the different impacts of PPI identified in this study and how they contribute to new knowledge	19-23
8c: Definition	Comment on the definition of PPI used (reported in the Background section) and whether or not you would suggest any changes	22
8d: Theoretical Underpinnings	Comment on any way your study adds to the theoretical development of PPI	23
8e: Context	Comment on how context factors influenced PPI in the study	19-23
8f: Process	Comment on how process factors influenced PPI in the study	19-23
8 g: Measurement and capture of PPI impact	If applicable, comment on how well PPI impact was evaluated or measured in the study	17-18
8 h: Economic assessment	If applicable, discuss any aspects of the economic cost or benefit of PPI, particularly any suggestions for future economic modelling	28
8i: Reflections/ critical perspective	Comment critically on the study, reflecting on the things that went well and those that did not, so that others can learn from this study	19-23

Reference:

1. Staniszewska S, Brett J, Simera I, Seers K, Mockford C, Goodlad S, et al. GRIPP2 reporting checklists: tools to improve reporting of patient and public involvement in research. Research Involvement & Engagement. 2017;3:13.
