# Supplementary material for: Needs led research: ensuring relevant research in two PhD projects within maternity care
Source: Res Involv Engagem. 2024 Sep 12;10:95. doi: 10.1186/s40900-024-00627-6 (PMC11391717; doi:10.1186/s40900-024-00627-6)
Supplement: Supplementary file 3 — Supplementary Material 3 [file 40900_2024_627_MOESM3_ESM.pdf]

Additional File 3: NLR-LINO

Search terms to identify and verify evidence gaps in NLR-LNO

Databases: The MEDLINE Database and the Cochrane Database of Systematic Reviews

Search date: September and October 2019

Limits: Reviews from the last 10 years

Question guiding the search: “What are the research needs/evidence gaps within the scope “labor induction”?

Search terms:

Labo*r AND induc* in title

Results from literature searches, papers included in NLR-LINO

Title	Authors	Year	Journal
A systematic review of implementing an elective labor induction policy.	Akinsipe DC, Villalobos LE, Ridley RT	2012	JOGNN - Journal of Obstetric, Gynecologic, & Neonatal Nursing.
Woman-centered care: Women's experiences and perceptions of induction of labor for uncomplicated post-term pregnancy: A systematic review of qualitative evidence	Akuamoah-Boateng J, Spencer R	2018	Midwifery
Oral misoprostol for induction of labour.	Alfirevic Z, Aflaifel N, Weeks A	2014	Cochrane Database of Systematic Reviews
Labour induction with prostaglandins: a systematic review and network meta-analysis.	Alfirevic Z, Keeney E, Dowswell T, Welton NJ, Dias S, Jones LV, Navaratnam K, Caldwell DM	2015	BMJ
Methods to induce labour: a systematic review, network meta-analysis and cost-effectiveness analysis.	Alfirevic Z, Keeney E, Dowswell T, Welton NJ, Medley N, Dias S, Jones LV, Caldwell DM	2016	BJOG: An International Journal of Obstetrics & Gynaecology
Which method is best for the induction of labour? A systematic review, network meta-analysis and cost-effectiveness analysis.	Alfirevic Z, Keeney E, Dowswell T, Welton NJ, Medley N, Dias S, Jones LV, Gyte G, Caldwell DM	2016	Health Technology Assessment

Intravenous oxytocin alone for cervical ripening and induction of labour.	Alfirevic Z, Kelly AJ, Dowswell T	2009	Cochrane Database of Systematic Reviews
Labor induction with intravaginal misoprostol compared with the dinoprostone vaginal insert: a systematic review and metaanalysis.	Austin SC, Sanchez-Ramos L, Adair CD	2010	American Journal of Obstetrics & Gynecology
Planned birth at or near term for improving health outcomes for pregnant women with pre-existing diabetes and their infants.	Biesty LM, Egan AM, Dunne F, Smith V, Meskell P, Dempsey E, Ni Bhuinneain GM, Devane D	2018	Cochrane Database of Systematic Reviews
Discontinuation of intravenous oxytocin in the active phase of induced labour.	Boie S, Glavind J, Velu AV, Mol BWJ, Uldbjerg N, de Graaf I, Thornton JG, Bor P, Bakker JJ	2018	Cochrane Database of Systematic Reviews
Planned early birth versus expectant management for women with preterm prelabour rupture of membranes prior to 37 weeks' gestation for improving pregnancy outcome.	Bond DM, Middleton P, Levett KM, van der Ham DP, Crowther CA, Buchanan SL, Morris J	2017	Cochrane Database of Systematic Reviews
Induction of labor: update and review.	Bonsack CF, Lathrop A, Blackburn M	2014	Journal of Midwifery & Women's Health.
Induction of labour at or near term for suspected fetal macrosomia.	Boulvain M, Irion O, Dowswell T, Thornton JG	2016	Cochrane Database of Systematic Reviews
When to Deliver Women with Diabetes in Pregnancy?	Caughey AB, Valent AM	2016	American Journal of Perinatology.
Meta-analysis of Foley catheter plus misoprostol versus misoprostol alone for cervical ripening.	Chen W, Xue J, Gaudet L, Walker M, Wen SW	2015	International Journal of Gynaecology & Obstetrics
A systematic review and network meta-analysis comparing the use of Foley catheters, misoprostol, and dinoprostone for cervical ripening in the induction of labour.	Chen W, Xue J, Wen SW	2016	BJOG: An International Journal of Obstetrics & Gynaecology
Planned early delivery versus expectant management for hypertensive disorders from 34 weeks gestation to term.	Cluver C, Novikova N, Koopmans CM, West HM	2017	Cochrane Database of Systematic Reviews
Induction of labour: Experiences of care and decision-making of women and clinicians	Coates D, Goodfellow A, Sinclair L	2019	Women & Birth: Journal of the Australian College of Midwives.
	Coates R, Cupples G, Scamell A, McCourt C	2019	Midwifery

Women's experiences of induction of labour: Qualitative systematic review and thematic synthesis			
Elective repeat caesarean section versus induction of labour for women with a previous caesarean birth.	Dodd JM, Crowther CA, Grivell RM, Deussen AR	2017	Cochrane Database of Systematic Reviews
Clinical trials during pregnancy: what has been done.	Dominguez V, Ramos N, Torrents A, Garcia D, Carne X	2012	European Journal of Clinical Pharmacology
Different methods for the induction of labour in outpatient settings.	Dowswell T, Kelly AJ, Livio S, Norman JE, Alfirevic Z	2010	Cochrane Database of Systematic Reviews
Breastfeeding Outcomes After Oxytocin Use During Childbirth: An Integrative Review.	Erickson EN, Emeis CL	2017	Journal of Midwifery & Women's Health.
Intravaginal misoprostol versus Foley catheter for labour induction: a meta-analysis.	Fox NS, Saltzman DH, Roman AS, Klauser CK, Moshier E, Rebarber A	2011	BJOG: An International Journal of Obstetrics & Gynaecology.
Nitric oxide donors for cervical ripening and induction of labour.	Ghosh A, Lattey KR, Kelly AJ	2016	Cochrane Database of Systematic Reviews
Risk of maternal, fetal and neonatal complications associated with the use of the transcervical balloon catheter in induction of labour: A systematic review.	Gommers JSM, Diederen M, Wilkinson C, Turnbull D, Mol BWJ	2017	European Journal of Obstetrics, Gynecology, & Reproductive Biology.
Current pharmacotherapy options for labor induction.	Hawkins JS, Wing DA	2012	Expert Opinion on Pharmacotherapy.
Vaginal misoprostol for cervical ripening and induction of labour.	Hofmeyr GJ, Gulmezoglu AM, Pileggi C	2010	Cochrane Database of Systematic Reviews
Mechanical methods for induction of labour.	Jozwiak M, Bloemenkamp KW, Kelly AJ, Mol BW, Irion O, Boulvain M	2012	Cochrane Database of Systematic Reviews
Ultrasound cervical length measurement in prediction of labor induction outcome.	Kehila M, Abouda HS, Sahbi K, Cheour H, Chanoufi MB	2016	Journal of Neonatal-Perinatal Medicine
Balloon catheters for induction of labor at term after previous cesarean section: a systematic review.	Kehl S, Weiss C, Rath W	2016	European Journal of Obstetrics, Gynecology, & Reproductive Biology.
Castor oil, bath and/or enema for cervical priming and induction of labour.	Kelly AJ, Kavanagh J, Thomas J	2013	Cochrane Database of Systematic Reviews
Timing induction of labour at 41 or 42 weeks? A closer look at time frames of comparison: A review.	Keulen JKJ, Bruinsma A, Kortekaas JC, van Dillen J, van der Post JAM, de Miranda E	2018	Midwifery

The Bishop score as a predictor of labor induction success: a systematic review.	Kolkman DG, Verhoeven CJ, Brinkhorst SJ, van der Post JA, Pajkrt E, Opmeer BC, Mol BW	2013	American Journal of Perinatology.
Is There a Place for Outpatient Preinduction Cervical Ripening?	Leopold B, Sciscione A	2017	Obstetrics & Gynecology Clinics of North America
Non-hormonal methods for induction of labour.	Lim CE, Ng RW, Xu K	2013	Current Opinion in Obstetrics & Gynecology
Effect of acupuncture on induction of labor.	Lim CE, Wilkinson JM, Wong WS, Cheng NC	2009	Journal of Alternative & Complementary Medicine
Induction of Labor and Cesarean: What is the True Relationship?	Little SE, Caughey AB	2015	Clinical Obstetrics & Gynecology.
Efficacy and safety of intravaginal misoprostol versus intracervical dinoprostone for labor induction at term: a systematic review and meta-analysis.	Liu A, Lv J, Hu Y, Lang J, Ma L, Chen W	2014	Journal of Obstetrics & Gynaecology Research.
Diagnostic accuracy of cervical elastography in predicting labor induction success: a systematic review and meta-analysis.	Londero AP, Schmitz R, Bertozzi S, Driul L, Fruscalzo A	2016	Journal of Perinatal Medicine
Women's experiences of postterm induction of labor: A systematic review of qualitative studies	Lou S, Hvidman L, Uldbjerg N, Neumann L, Jensen TF, Haben JG, Carstensen K	2018	Birth
Induction of labour for suspected macrosomia at term in non-diabetic women: a systematic review and meta-analysis of randomized controlled trials.	Magro-Malosso ER, Saccone G, Chen M, Navathe R, Di Tommaso M, Berghella V	2017	BJOG: An International Journal of Obstetrics & Gynaecology.
Evaluation of a Transcervical Foley Catheter as a Source of Infection: A Systematic Review and Meta-analysis.	McMaster K, Sanchez-Ramos L, Kaunitz AM	2015	Obstetrics & Gynecology.
Induction of labour for improving birth outcomes for women at or beyond term.	Middleton P, Shepherd E, Crowther CA	2018	Cochrane Database of Systematic Reviews
Planned early birth versus expectant management (waiting) for prelabour rupture of membranes at term (37 weeks or more).	Middleton P, Shepherd E, Flenady V, McBain RD, Crowther CA	2017	Cochrane Database of Systematic Reviews
Use of labour induction and risk of cesarean delivery: a systematic review and meta-analysis.	Mishanina E, Rogozinska E, Thatthi T, Uddin-Khan R, Khan KS, Meads C	2014	CMAJ Canadian Medical Association Journal
Methods of induction of labour: a systematic review.	Mozurkewich EL, Chilimigras JL, Berman	2011	BMC Pregnancy & Childbirth

	DR, Perni UC, Romero VC, King VJ, Keeton KL		
The association between the regular use of preventive labour induction and improved term birth outcomes: findings of a systematic review and meta-analysis.	Nicholson JM, Kellar LC, Henning GF, Waheed A, Colon-Gonzalez M, Ural S	2015	BJOG: An International Journal of Obstetrics & Gynaecology.
Methods of classification for women undergoing induction of labour: a systematic review and novel classification system.	Nippita TA, Khambalia AZ, Seeho SK, Trevena JA, Patterson JA, Ford JB, Morris JM, Roberts CL	2015	BJOG: An International Journal of Obstetrics & Gynaecology.
Hypnosis for induction of labour.	Nishi D, Shirakawa MN, Ota E, Hanada N, Mori R	2014	Cochrane Database of Systematic Reviews
Uterine rupture during trial of labor: controversy of induction's methods.	Ophir E, Odeh M, Hirsch Y, Bornstein J	2012	Obstetrical & Gynecological Survey.
The role of sonographic cervical length in labor induction at term.	Papillon-Smith J, Abenhaim HA	2014	Journal of Clinical Ultrasound.
Labor Induction Techniques: Which Is the Best?	Penfield CA, Wing DA	2017	Obstetrics & Gynecology Clinics of North America
Labor induction: a review of current methods.	Ramirez MM	2011	Obstetrics & Gynecology Clinics of North America
Antibiotics prior to amniotomy for reducing infectious morbidity in mother and infant.	Ray A, Ray S	2014	Cochrane Database of Systematic Reviews
Discontinuing Oxytocin Infusion in the Active Phase of Labor: A Systematic Review and Meta-analysis.	Saccone G, Ciardulli A, Baxter JK, Quinones JN, Diven LC, Pinar B, Maruotti GM, Martinelli P, Berghella V	2017	Obstetrics & Gynecology.
A safety review of medications used for labour induction.	Sheibani L, Wing DA	2018	Expert Opinion on Drug Safety.
Isolated Oligohydramnios at Term as an Indication for Labor Induction: A Systematic Review and Meta-Analysis.	Shrem G, Nagawkar SS, Hallak M, Walfisch A	2016	Fetal Diagnosis & Therapy
Acupuncture or acupressure for induction of labour.	Smith CA, Armour M, Dahlen HG	2017	Cochrane Database of Systematic Reviews
Acupuncture for induction of labour.	Smith CA, Crowther CA, Grant SJ	2013	Cochrane Database of Systematic Reviews
Misoprostol vaginal insert for induction of labor: a delivery system with accurate dosing and rapid discontinuation.	Stephenson ML, Hawkins JS, Powers BL, Wing DA	2014	Women's health.

Comparison of outcomes between induction of labor and spontaneous labor for term breech - A systemic review and meta analysis.	Sun W, Liu F, Liu S, Gratton SM, El-Chaar D, Wen SW, Chen D	2018	European Journal of Obstetrics, Gynecology, & Reproductive Biology
The Bishop Score as a determinant of labour induction success: a systematic review and meta-analysis.	Teixeira C, Lunet N, Rodrigues T, Barros H	2012	Archives of Gynecology & Obstetrics.
Induction of Labor Using a Foley Catheter or Misoprostol: A Systematic Review and Meta-analysis.	Ten Eikelder ML, Mast K, van der Velden A, Bloemenkamp KW, Mol BW	2016	Obstetrical & Gynecological Survey.
Vaginal prostaglandin (PGE2 and PGF2a) for induction of labour at term.	Thomas J, Fairclough A, Kavanagh J, Kelly AJ	2014	Cochrane Database of Systematic Reviews
Foley catheter balloon vs locally applied prostaglandins for cervical ripening and labor induction: a systematic review and metaanalysis.	Vaknin Z, Kurzweil Y, Sherman D	2010	American Journal of Obstetrics & Gynecology
Transvaginal sonographic assessment of cervical length and wedging for predicting outcome of labor induction at term: a systematic review and meta-analysis.	Verhoeven CJ, Opmeer BC, Oei SG, Latour V, van der Post JA, Mol BW	2013	Ultrasound in Obstetrics & Gynecology.
Pharmacological and mechanical interventions for labour induction in outpatient settings.	Vogel JP, Osoti AO, Kelly AJ, Livio S, Norman JE, Alfirevic Z	2017	Cochrane Database of Systematic Reviews
Delivery at Term: When, How, and Why.	Walker KF, Thornton JG	2018	Clinics in Perinatology
Controlled-release dinoprostone insert versus Foley catheter for labor induction: a meta-analysis.	Wang H, Hong S, Liu Y, Duan Y, Yin H	2016	Journal of Maternal-Fetal & Neonatal Medicine
Efficacy and safety of misoprostol compared with the dinoprostone for labor induction at term: a meta-analysis.	Wang L, Zheng J, Wang W, Fu J, Hou L	2016	Journal of Maternal-Fetal & Neonatal Medicine
Methods of term labour induction for women with a previous caesarean section.	West HM, Jozwiak M, Dodd JM	2017	Cochrane Database of Systematic Reviews
Pharmacotherapy options for labor induction.	Wing DA, Sheibani L	2015	Expert Opinion on Pharmacotherapy.
Double-balloon versus single-balloon catheter for cervical ripening and labor induction: A systematic review and meta-analysis.	Yang F, Huang S, Long Y, Huang L	2018	Journal of Obstetrics & Gynaecology Research

Maternal and fetal best interests in day-to-day obstetrics.	Yeo GS, Lim ML	2011	Annals of the Academy of Medicine, Singapor
Misoprostol versus prostaglandin E2 gel for labor induction in premature rupture of membranes after 34 weeks of pregnancy.	Zhang Y, Wang J, Yu Y, Xie C, Xiao M, Ren L	2015	International Journal of Gynaecology & Obstetrics
