# Supplementary material for: Needs led research: ensuring relevant research in two PhD projects within maternity care
Source: Res Involv Engagem. 2024 Sep 12;10:95. doi: 10.1186/s40900-024-00627-6 (PMC11391717; doi:10.1186/s40900-024-00627-6)
Supplement: Supplementary file 4 — Supplementary Material 4 [file 40900_2024_627_MOESM4_ESM.pdf]

Additional File 4: NLR-LISTEN

Search terms to identify and verify evidence gaps in NLR-LISTEN

Databases: The MEDLINE Database and the Cochrane Database of Systematic Reviews

Search date: September and October 2019

Limits: Reviews from the last 10 years

Question guiding the search: “What are the research needs/evidence gaps within the scope “Fetal monitoring in low-risk deliveries”?

Search terms

Population	Intervention	Context
Low risk	heart auscultation	Intrapartum
Lowrisk	fetal monitoring	labour
Low-risk	foetal monitoring	labor
Normal	fetal surveillance	delivery maternity care
	foetal surveillance	
	perinatal care	
	fetal heartrate	
	foetal heartrate	
	fetal monitoring method	
	foetal monitoring method	
	fetal well-being	
	fetal wellbeing	
	fetal assessment	
	doppler	
	pinard stethoscope	
	pinard	
	intermittent auscultation	
	IA	

Results from literature searches, papers included in NLR-LISTEN

Title	Authors	Year	Journal
Cardiotocography versus intermittent auscultation of fetal heart on admission to labour ward for assessment of fetal wellbeing.	Devane D, Lalor J, Daly S, McGuire W, Cuthbert A, Smith V.	2017	The Cochrane Library
Intermittent auscultation (IA) of fetal heart rate in labour for fetal well-being.	Martis R, Emilia O, Nurdiati D, Brown J.	2017	The Cochrane Library
Intermittent auscultation fetal monitoring during labour: A systematic scoping review to identify methods, effects, and accuracy.	Blix E, Maude R, Hals E, Kisa S, Karlsen E, Nohr EA, de Jonge A, Lindgren H, Downe S, Reinar LM, Foureur M, Pay ASD, Kaasen A.	2019	PLOS ONE
Vibroacoustic stimulation for fetal assessment in labour in the presence of a nonreassuring fetal heart rate trace.	Christine E East, Rebecca MD Smyth, Leo R Leader, Naomi E Henshall, Paul B Colditz, Rosalind Lau, Kelvin H Tan	2014	The Cochrane Library
Interrelations Between Four Antepartum Obstetric Interventions and Cesarean Delivery in Women at Low Risk: A Systematic Review and Modeling of the Cascade of Interventions	Michel Rossignol, MD, MSc, FRCPC, Nils Chaillet, PhD, Faiza Boughrassa, MD, MSc, and Jean-Marie Moutquin, MD, MSc, FRCSC	2014	Birth: Issues in Perinatal Care
Women's experiences of continuous fetal monitoring - a mixed-methods systematic review	Crawford A, Hayes D, Johnstone ED, Heazell AEP.	2017	Acta Obstetricai et Gynecologica Scandinavia
Professionals' views of fetal monitoring during labour: a systematic review and thematic analysis.	Smith V, Begley CM, Clarke M, Devane D.	2012	BMC Pregnancy and Childbirth
Fetal movements as a predictor of health.	Jonathan Lai Niamh C. Nowlan Ravi Vaidyanathan Caroline J. Shaw Christoph C. Lees	2016	Acta Obstetricai et Gynecologica Scandinavia
